# Dimerization of the BAR domain–containing protein FAM92A modulates lipid binding and interaction with CBY1

**DOI:** 10.1016/j.jbc.2025.110346

**Published:** 2025-06-06

**Authors:** Xiaohan Xu, Jing Ren, Jianchao Li

**Affiliations:** Innovation Centre of Ministry of Education for Development and Diseases, School of Medicine, South China University of Technology, Guangzhou, China

**Keywords:** cilia, lipid-binding protein, crystal structure, FAM92A, CBY1

## Abstract

BAR (Bin/Amphiphysin/Rvs) domain proteins drive membrane remodeling critical for cellular processes like ciliogenesis and organelle morphology. FAM92A (family with sequence similarity 92A), a classical BAR protein, regulates ciliary assembly, mitochondrial ultrastructure, and neuronal membrane dynamics, yet its molecular mechanisms remain elusive. Here, we determined the 2.2 Å crystal structure of the mouse FAM92A BAR domain, revealing an antiparallel, crescent-shaped homodimer. Structure-guided mutagenesis revealed that positively charged clusters on the concave surface are critical for lipid binding and identified residues essential for dimerization. We further demonstrated that FAM92A BAR directly binds the N-terminal region of Chibby1 (CBY1), a ciliary protein, with their respective dimerizations synergistically enhancing affinity. These findings elucidate the structural basis of FAM92A’s membrane remodeling and CBY1 interaction, providing a molecular framework for its function in ciliogenesis and suggesting broader implications for FAM92 family proteins.

The BAR (Bin/Amphiphysin/Rvs) domain superfamily proteins are central regulators of dynamic membrane remodeling in cells (1, 2). These proteins are characterized by a conserved BAR domain, a rigid α-helical bundle formed through dimerization, adopting a crescent shape (3, 4). The concave or convex surfaces of this domain are enriched with positively charged residues, enabling electrostatic interactions with negatively charged membrane phospholipids (5, 6, 7, 8). Based on their curvature properties, BAR domains are classified into three major subtypes: classical BAR (including the N-BAR subclass) and F-BAR domains, which induce membrane invagination *via* their concave surfaces, and the inversely curved I-BAR domain, which promotes membrane protrusions through its convex surface (9, 10, 11, 12). The BAR domain exhibits multiple functions, including the induction, stabilization, and sensing of membrane curvature, allowing them to play a critical role in signal-structure coupling during processes, such as vesicle trafficking, organelle shaping, and cell motility (2, 13, 14, 15, 16). A detailed understanding of the structural features of individual family members is essential for elucidating their diverse biological roles.

FAM92A (also known as BARMR1 or CIBAR1), first cloned from human testis, is a gene encoding a BAR domain–containing protein and belongs to the FAM92 (family with sequence similarity 92) family alongside FAM92B (CIBAR2) (17, 18). Knockdown of FAM92A in RPE1 cells or its knockout in NIH-3T3 cells results in pronounced ciliogenesis defects (18, 19). In addition, FAM92A has been shown to localize to the matrix side of the mitochondrial inner membrane, where its absence severely disrupts mitochondrial morphology and ultrastructure, and consequently impairs organelle bioenergetics (20). Animal model studies reveal that homozygous Fam92a knockout mice (Fam92a^−/−^) exhibit significant developmental abnormalities, including metatarsal defects, osteomas, axial polydactyly, and deltoid tuberosity anomalies of the humerus (21). Furthermore, FAM92A knockout mice display embryonic lethality because of left-right asymmetry defects, pancreatic degeneration, altered glucose tolerance, and ciliary deficiencies (22), as well as altered brain morphology, impaired synaptic transmission and plasticity, and age-related cognitive deficits (23). In *Xenopus*, both knockdown and overexpression of FAM92A induce developmental anomalies (24). Moreover, FAM92A deficiency in mice and *Drosophila* is associated with reduced male fertility (25, 26). In humans, FAM92A aberrations are linked to multiple disorders, including Nablus mask–like facial syndrome (27), cerebellar hypoplasia, and autism (21). FAM92A may also regulate tumor proliferation, survival, and migration, with its expression levels correlating with overall survival in acute myeloid leukemia patients (28) and aberrant expression observed in penile cancer (29).

In ciliogenesis, FAM92A plays a critical role through its interaction with Chibby1 (CBY1). The two proteins colocalize at the mother centriole of the ciliary basal body/transition zone, forming a functional complex, with the BAR domain of FAM92A mediating its binding to CBY1 (18, 30). Similarly, in *Drosophila*, Cby interacts with Fam92 to regulate transition zone assembly (25), highlighting the evolutionary conservation of this interaction. Intriguingly, testis-specific CBY3 has also been reported to interact with FAM92A to modulate spermatogenesis (26), underscoring the broader relevance of this interaction within the CBY family. However, the precise molecular mechanisms underlying the FAM92A–CBY1 interaction remain to be fully elucidated.

In this study, employing biochemical and structural biology approaches, we demonstrated that the BAR domain of mouse FAM92A forms a dimer and resolved its crystal structure at 2.2 Å resolution. Structural analysis further delineated the key interface responsible for dimerization as well as the membrane-binding interface and the critical amino acid residues involved. We also characterized the interaction between FAM92A and CBY1 in detail, revealing a synergistic interplay between the BAR domain–mediated dimer of FAM92A and the coiled-coil (CC) dimer of CBY1’s C terminus, which significantly enhances their binding affinity. These findings provide a molecular and structural framework for understanding the roles of FAM92A and CBY1 in ciliogenesis.

## Results

### The BAR domain of FAM92A forms homodimers in solution

**Figure 1:**
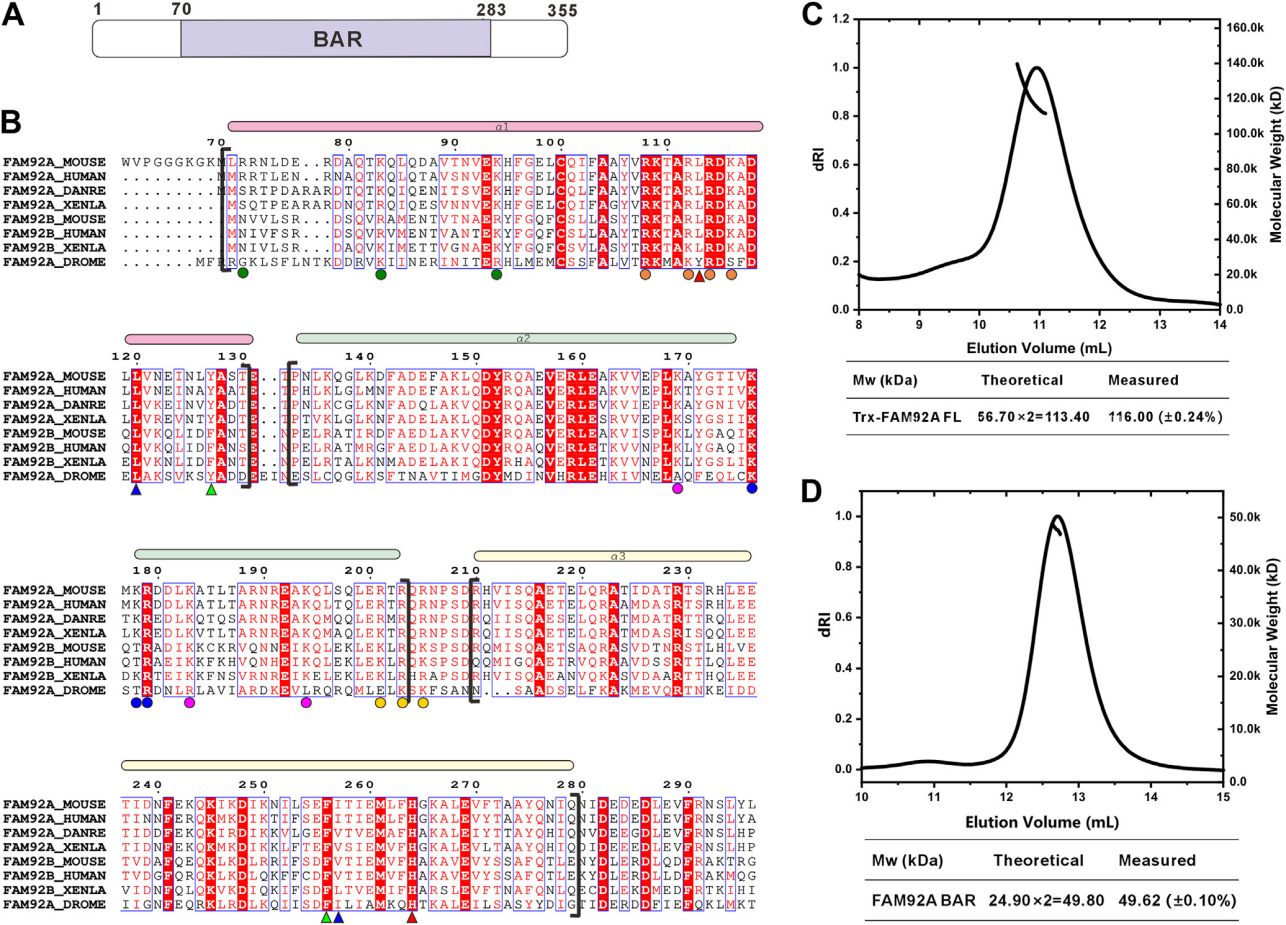
**Homodimerization of the FAM92A BAR domain**. *A*, schematic of mouse FAM92A domain organization, highlighting the BAR domain (residues 70–283). *B*, multiple sequence alignment of the FAM92A and FAM92B BAR domain across species (MOUSE: *Mus musculus*; HUMAN: *Homo sapiens*; DANRE: *Danio rerio*; XENLA: *Xenopus laevis*; and DROME: *Drosophila melanogaster*). The alignment, generated by ClustalX and ESPript, depicts helical structures, highlighting strictly conserved residues with *white boxes* on a *red background* and highly conserved residues with *red boxes* on a *white background*. The *triangles* represent the key residues for dimerization (*e*.*g*., L120, I257), and the *dots* represent the key positively charged residues for lipid binding (*e*.*g*., R108, K109). Colors correspond to curves of SEC–MALS in Figures S2 and S4*B*. *C* and *D*, SEC–MALS profiles of Trx-tagged full-length FAM92A (*C*) and untagged FAM92A BAR domain (*D*), confirming homodimeric states with molecular weights of ∼116 kDa and ∼49.6 kDa, respectively. The measured molecular weights are expressed as Mn (±x%). BAR, Bin/Amphiphysin/Rvs; FAM92A, family with sequence similarity 92A; SEC–MALS, size-exclusion chromatography coupled with multiangle light scattering.

Mouse FAM92A possesses a central BAR domain (amino acids 70–283) flanked by unstructured regions at both the N and C termini (Fig. 1*A*). Multiple sequence alignment showed the striking evolutionary conservation of the BAR domain of FAM92A among different species (Fig. 1*B*). BAR domains are well known to function as dimers. To examine the oligomeric state of FAM92A BAR domain, we first expressed recombinant full-length FAM92A (FAM92A FL) and the isolated BAR domain (FAM92A BAR) proteins in *Escherichia coli*. Following purification under physiological salt conditions (100 mM NaCl, pH 7.5), size-exclusion chromatography coupled with multiangle light scattering (SEC–MALS) assay revealed that Trx-tagged FAM92A FL eluted as a monodisperse peak with a molecular weight (Mw) of 116 kDa, approximately twice the theoretical Mw (56.7 kDa), confirming its homodimeric assembly in solution (Fig. 1*C*). Similarly, the untagged BAR domain exhibited consistent dimeric behavior, with a measured Mw of 49.62 kDa (theoretical dimer Mw: 49.8 kDa), indicating that the BAR domain alone is sufficient to mediate dimerization (Fig. 1*D*).

### The overall structure of the FAM92A BAR domain

To gain deeper structural insights into the FAM92A BAR domain, we pursued to determine its atomic structure using X-ray crystallography. After optimization, we obtained crystals diffracting to a resolution of 2.2 Å (Table 1). The structure was solved by molecular replacement, employing the AlphaFold2-predicted model as a search template (31). With the exception of short segments at the N and C termini, most amino acid residues were well resolved in the final electron density maps. Each asymmetric unit contains one FAM92A BAR molecule, with the homodimer generated by a crystallographic twofold symmetry axis.

**Table 1:** Statistics of X-ray crystallographic data collection and model refinement

Parameters	Values
Data collection	
Datasets	FAM92A_Bar
Space group	*P3*_*1*_2 1
Wavelength (Å)	0.97923
Unit cell parameters (Å)	*a* = *b* = 132.27, *c* = 67.76 *α* = *β* = 90° *γ* = 120°
Resolution range (Å)	57.27–2.21 (2.27–2.21)
No. of unique reflections	34,469 (2526)
Redundancy	19.7 (20.1)
I/σ	17.5 (1.6)
Completeness (%)	100 (99.9)
*R*_merge_^a^ (%)	18.9 (243.9)
CC_1/2_^b^	0.999 (0.713)
Structure refinement	
Resolution (Å)	58.320–2.210 (2.289–2.210)
*R*_cryst_^c^/*R*_free_^d^ (%)	18.24/21.45 (23.03/30.83)
RMSD bonds (Å)/angles (°)	0.002/0.43
Average *B*-factor (Å^2^)^e^	54.64
No. of atoms	
Protein atoms	1920
Water	156
Ligands	0
No. of reflections	
Working set	32,260 (3196)
Test set	1762 (188)
Ramachandran plot regions^d^	
Favored (%)	98.61
Allowed (%)	1.39
Outliers (%)	0

Numbers in parentheses represent the value for the highest resolution shell.

a *R*_merge_ = Σ|*I*_*i*_ - <*I*>|/Σ*I*_*i*_, where *I*_*i*_ is the intensity of measured reflection and <*I*> is the mean intensity of all symmetry-related reflections.

b CC_1/2_ were defined by Karplus and Diederichs (43).

c *R*_cryst_ = Σ||*F*_calc_| – |*F*_obs_||/Σ*F*_obs_, where *F*_obs_ and *F*_calc_ are observed and calculated structure factors.

d *R*_free_ = Σ_T_||*F*_calc_| – |*F*_obs_||/Σ*F*_obs_, where T is a test dataset of about 5% of the total unique reflections randomly chosen and set aside prior to refinement.

e *B*-factors and Ramachandran plot statistics are calculated using MOLPROBITY (42).

**Figure 2:**
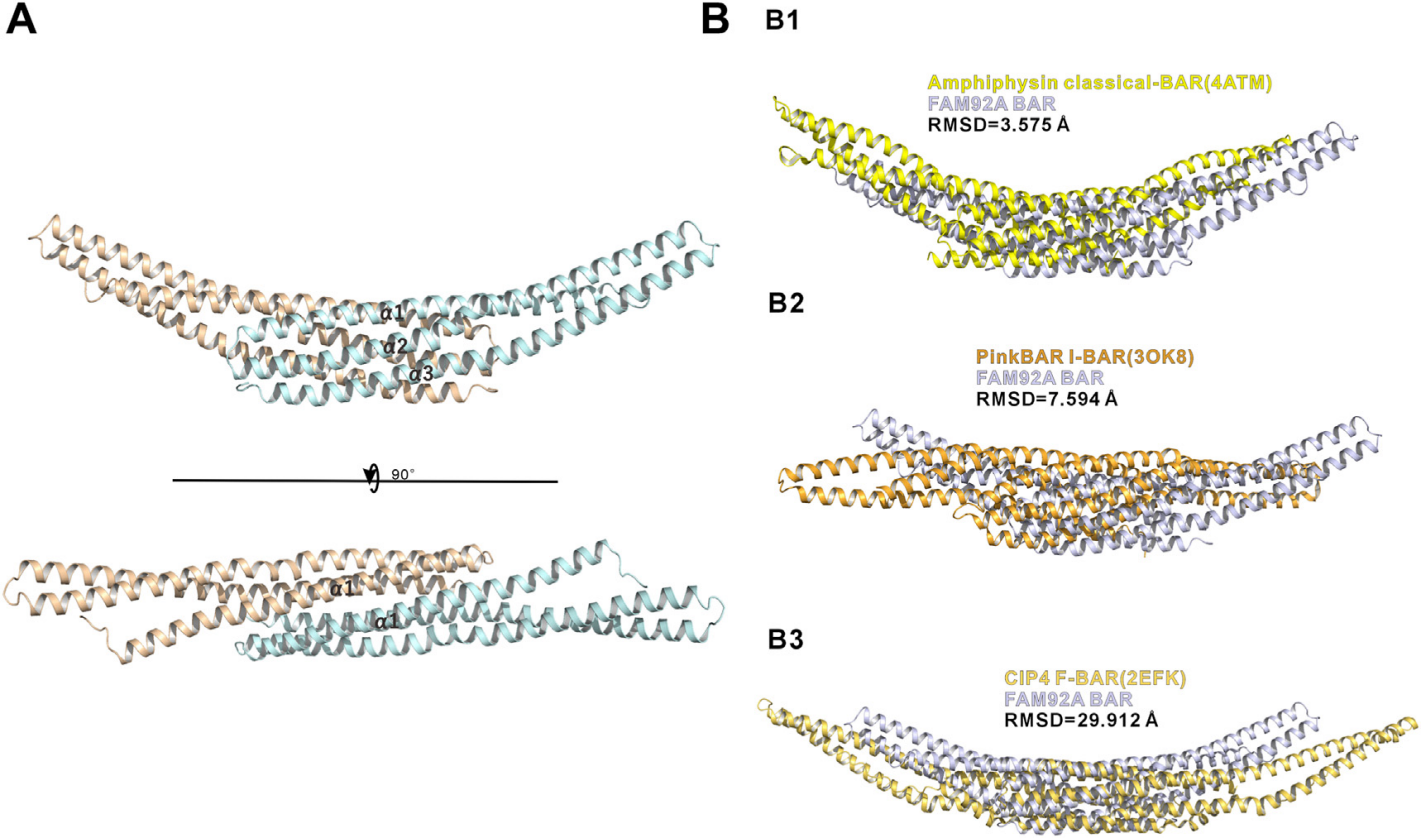
**Crystal structure of the FAM92A BAR domain**. *A*, Ribbon diagram of the FAM92A BAR homodimer at 2.2 Å resolution, showing the antiparallel, crescent-shaped arrangement. *Top*, side view highlighting the dimer interface; *bottom*, concave surface view highlighting the potential lipid-binding surface. *B*, structural superposition of FAM92A BAR with representative BAR domains: amphiphysin (classical BAR, PDB code: 4ATM, B1), PinkBAR (I-BAR, PDB: 3OK8, B2), and CIP4 (F-BAR, PDB code: 2EFK, B3), highlighting closest similarity to the classical BAR subtype with an RMSD of 3.575 Å. Superpositions were performed using the cealign algorithm in PyMOL, aligning the dimeric FAM92A BAR domain with each comparator dimeric BAR structure. BAR, Bin/Amphiphysin/Rvs; FAM92A, family with sequence similarity 92A; PDB, Protein Data Bank.

Each monomer consists of three extended α-helices connected by loops, featuring a characteristic kink in α2 (residues 160–170) induced by the conserved Pro167. Two monomers associate to form a crescent-shaped homodimer, with their C termini in close proximity (Fig. 2*A*). Structural comparison of the FAM92A BAR dimer with other types of BAR domain revealed the closest similarity to the classical BAR domain of amphiphysin (Protein Data Bank [PDB] code: 4ATM), with an RMSD of 3.575 Å (Fig. 2*B1*). In contrast, alignment with the F-BAR domain of CIP4 (PDB code: 2EFK) (8) and the I-BAR domain of PinkBAR (PDB code: 3OK8) (32) yielded higher RMSD values of 29.912 Å and 7.594 Å, respectively (Fig. 2, *B*2 and *B*3). These analyses classify FAM92A BAR as a member of the classical BAR domain subfamily.

While our work was in progress, the structure of human FAM92A1 BAR was reported (23). We, therefore, compared our mouse FAM92A BAR structure with its human counterpart, finding that the two structures align closely (RMSD of 0.519 Å), underscoring their high structural conservation (Fig. S1).

### The concave surface of FAM92A BAR domain dimer mediates membrane binding

**Figure 3:**
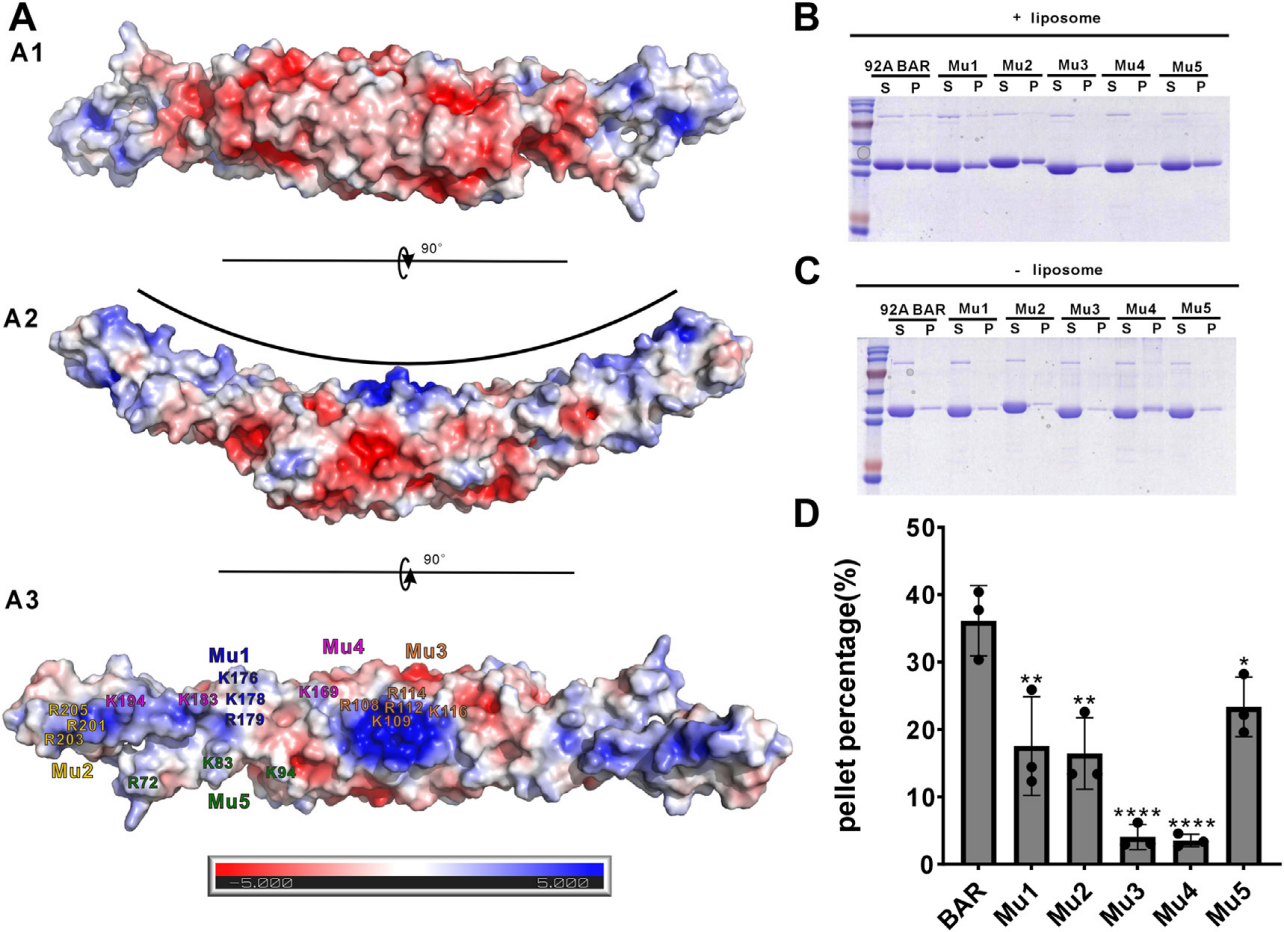
**Electrostatic features and membrane binding of the FAM92A BAR domain**. *A*, electrostatic surface potential of the FAM92A BAR homodimer, highlighting positively charged clusters on the concave membrane–binding face. Views: convex surface (A1), side view (A2), and concave surface (A3) with key lipid-binding residues. Mu1: K176, K178, and R179; Mu2: R201, R203, and R205; Mu3: R108, K109, R112, R114, and K116; Mu4: K169, K183, and K194; Mu5: R72, K83, and K94. Colors correspond to SEC–MALS curves in Figure S2. The charge potential surface was calculated by the APBS module embedded in PyMOL and contoured at ±5 kT/e. *B* and *C*, representative SDS-PAGE results showing the results of FAM92A BAR and its positively charged variants in binding to brain liposomes. In each assay, 10 μM proteins were mixed with (*B*) or without (*C*) 2 mg/ml brain liposomes in assay buffer. After high-speed centrifugation, the supernatant (S) and pellet (P) fractions were analyzed by SDS-PAGE with Coomassie *blue* staining. Each experiment was repeated three times. *D*, quantification of pellet fractions from brain liposome–binding assays, showing reduced binding for mutants. Pellet percentages were calculated as (P_+liposome/[P_+liposome + S_+liposome]) × 100%, without subtracting protein-only controls because of negligible nonspecific pelleting. Data are mean ± SD. Statistical significance was determined by one-way ANOVA with Dunnett’s test. ns, *p* > 0.05; ∗*p* < 0.05; ∗∗∗*p* < 0.001; ∗∗∗∗*p* < 0.0001. BAR, Bin/Amphiphysin/Rvs; FAM92A, family with sequence similarity 92A; SEC–MALS, size-exclusion chromatography coupled with multiangle light scattering.

BAR domains are well recognized for their capacity to bind negatively charged membranes. To investigate this property in FAM92A, we calculated the electrostatic potential surface of the newly determined FAM92A BAR dimer structure. The analysis reveals a distinct charge distribution: the concave surface is predominantly positively charged, whereas the convex surface exhibits a negative charge (Fig. 3*A*). On the concave face, positive charges cluster into several distinct regions. The most intense positive potential is located centrally, contributed by positively charged residues (R108, K109, R112, R114, and K116) from the dimer-forming α1 helices (denoted as region 1). Another prominent positively charged region (referred to as region 2) is observed at the two distal ends, primarily driven by R201, R203, and R205. In addition, three peripheral regions of positive charge were identified: region 3, comprising K176, K178, and R179; region 4, formed by K169, K183, and K194; and region 5, consisting of R72, K83, and K94 (Fig. 3*A*3). Multiple sequence alignment revealed that the positively charged lipid-binding residues of the FAM92A BAR domain are highly conserved across species and in FAM92B (Fig. 1*B*). These clustered positive charges on the concave surface likely facilitate interactions with negatively charged lipids.

To assess the functional significance of the identified positively charged regions, we individually mutated the positively charged residues in each region to glutamic acid and evaluated their impact on membrane-binding capacity using a brain liposome–based sedimentation assay with purified recombinant proteins (Fig. 3, *B* and *C*). As a control, SEC–MALS experiments confirmed that all mutant proteins retained their dimeric state in solution, indicating that these mutations do not disrupt the formation of the concave surface (Fig. S2). Consistent with previous studies and the established properties of BAR domains, a substantial fraction of WT FAM92A BAR is cosedimented with brain liposomes, confirming its membrane-binding capability (Fig. 3, *B* and *C*). In line with our aforementioned analysis, mutating the residues in region 1 (R108, K109, R112, R114, and K116) reduced the amount of copelleted protein by approximately 90% compared with WT, underscoring the critical role of this central region in lipid binding. Similarly, mutations in region 4 (K169, K183, and K194) resulted in ∼90% reduction, potentially because of the broader span of mutations designed for this region. In contrast, mutations in the remaining three regions led to more moderate reductions, ranging from approximately one-third to one-half of the pelleted protein relative to WT (Fig. 3, *B*–*C*). Collectively, these results demonstrate that multiple positively charged regions on the concave surface of the FAM92A BAR dimer contribute to its membrane-binding function.

### The detailed interactions mediated FAM92A BAR domain dimerization

**Figure 4:**
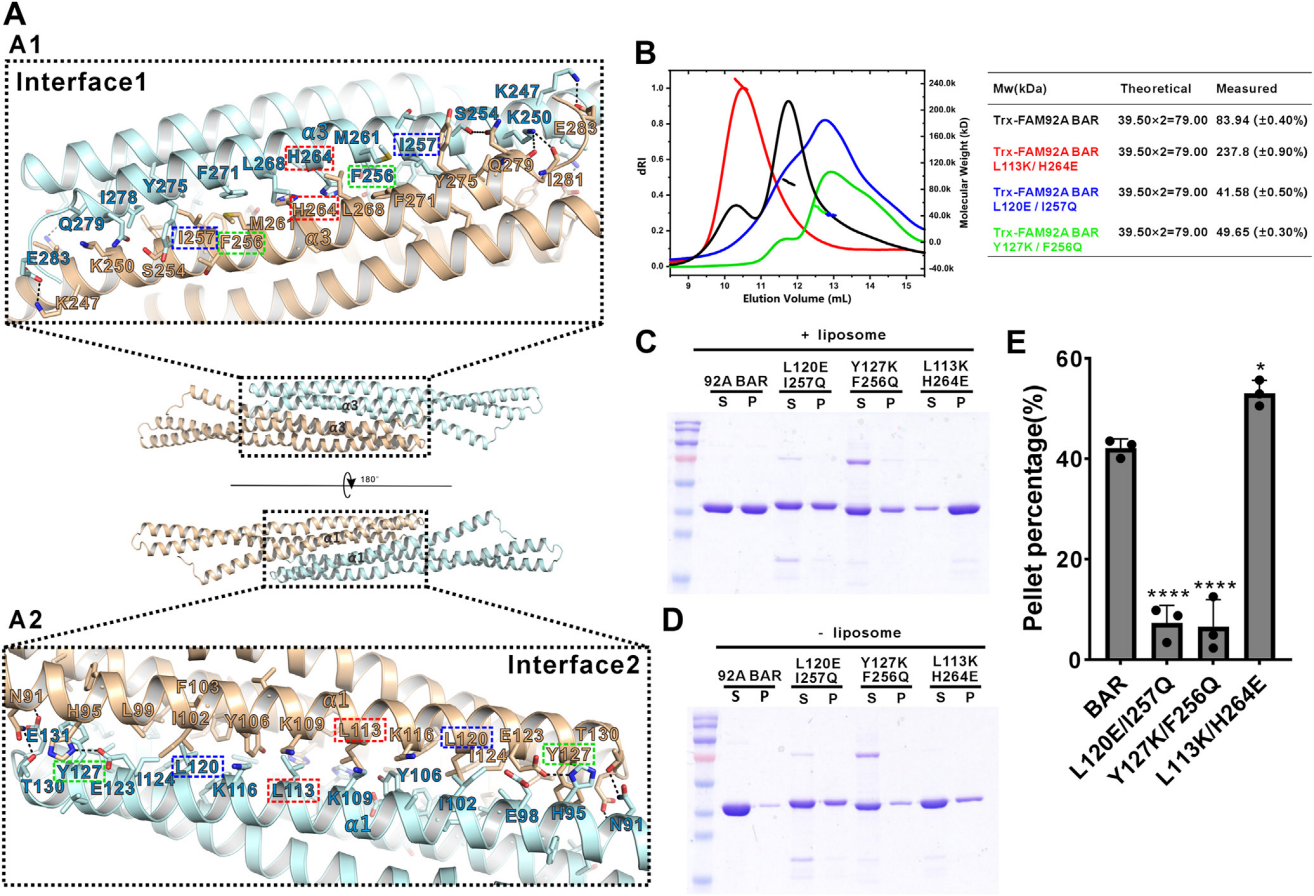
**Dimerization interface and membrane-binding role of FAM92A BAR domain**. *A*, structure of the mouse FAM92A BAR homodimer, showing two dimer interfaces: interface 1 (convex surface, α3 helices; A1) and nterface 2 (concave surface, α1 helices; A2). Residues responsible for the dimer formation are highlighted with *hollow boxes*. The colors correspond to SEC–MALS curves in *B*. *B*, SEC–MALS profiles of FAM92A BAR mutants (L120K/I257Q, Y127K/F256Q, and L113K/H264E), revealing disrupted dimerization or oligomerization. *C* and *D*, representative SDS-PAGE results showing the results of FAM92A BAR and its dimer-disrupting variants in binding to brain liposomes. Similar procedure is described in Figure 3, *B* and *C*. (*C*): with liposome; (*D*) without liposome. *E*, quantification of pellet fractions from brain liposome-binding assays, showing reduced or increased binding for mutants. Pellet percentages were calculated as ([P_+liposome − P_-liposome]/[P_+liposome + S_+liposome]) × 100%, after subtracting the values of protein-only controls to correct for nonspecific pelleting. Data are mean ± SD. Statistical significance was determined by one-way ANOVA with Dunnett’s test. ns, *p* > 0.05; ∗*p* < 0.05; ∗∗∗*p* < 0.001; ∗∗∗∗*p* < 0.0001. BAR, Bin/Amphiphysin/Rvs; FAM92A, family with sequence similarity 92A; SEC–MALS, size-exclusion chromatography coupled with multiangle light scattering.

The dimer is the principal functional unit of BAR domains. Like other BAR domains, the FAM92A BAR domain adopts an antiparallel dimeric arrangement, with the two monomers oriented oppositely along their long axes, forming a symmetric, crescent-shaped structure stabilized by a crystallographic twofold rotation axis (Fig. 2*A*). This dimerization buries an extensive interface of ∼2700 Å^2^. The interface can be broadly divided into two distinct regions: interface 1, on the convex surface, involves antiparallel α3 helices, whereas interface 2, on the concave surface, comprises antiparallel α1 helices (Fig. 4*A*). Both interfaces are predominantly stabilized by hydrophobic interactions. In interface 1, residues L253, F256, I257, M261, H264, L268, F271, Y275, and I278 from α3 create an antiparallel hydrophobic zipper with α3’. In interface 2, residues L99, I102, F103, Y106, K109, L113, K116, L120, I124, and Y127 from α1 form hydrophobic contacts with their counterparts on the opposing α1′ helix. Hydrophobic residues at the α2 N terminus (L139, F142, and F146) are also buried deeply within the interface, enhancing structural stability. In addition, hydrogen bonds (E123–H95, N91–T130, S254–Q279 side chains; K250 side chain to Q279/I281 main-chain carbonyls) and salt bridges (E131–K245, E283–K247) further reinforce the dimeric assembly (Fig. 4, *A*1 and *A*2).

To validate the structural findings, we introduced mutations to disrupt key hydrophobic interactions within the FAM92A BAR domain dimer. Mutating L120 to the negatively charged glutamic acid, together with I257 to the polar glutamine, abolished dimer formation, as shown by SEC–MALS analysis (Fig. 4*B*). Similarly, substituting Y127 with the positively charged lysine and F256 with glutamine also eliminated dimerization (Fig. 4*B*). Interestingly, mutating L113 to lysine and H264 to glutamic acid destabilized the dimer, yielding higher Mw oligomers, likely because of nonspecific aggregation (Fig. 4*B*). Sequence alignment showed that key dimerization residues of the FAM92A BAR domain are highly conserved across as well as in FAM92B, further underscoring the critical role of the BAR domain’s dimeric structure in its function (Fig. 1*B*).

We next assessed the dimer’s role in lipid binding using liposome cosedimentation assays. Both the dimer-disrupting L120E/I257Q and Y127K/F256Q mutants exhibited severely reduced lipid binding compared with WT (Fig. 4, *C–E*). In contrast, the oligomer-forming L113K/H264E mutant slightly increased the amount of sedimented proteins relative to WT, possibly because of nonspecific liposome interactions (Fig. 4, *C–E*).

Taken together, the high-resolution structure of FAM92A BAR elucidated the structural basis for dimerization, allowing targeted disruption and confirming dimerization’s critical role in membrane binding.

### FAM92A BAR domain can directly bind to CBY1

**Figure 5:**
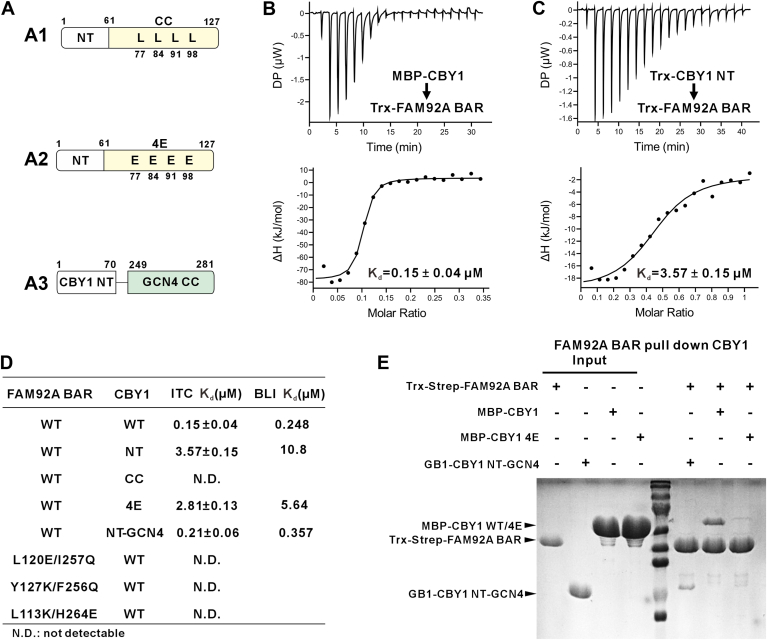
**Biochemical analysis of FAM92A BAR and CBY1 interaction**. *A*, schematics of mouse CBY1 and its different constructs. A1: CBY1 WT; A2: CBY1 4E (L77E, L84E, L91E, and L98E); A3: CBY1 NT-GCN4, fusing yeast GCN4 CC (amino acids 249–281) to the C-terminal region of CBY1 N-terminal (NT). NT and coiled-coil (CC) regions are shown, with 4E mutation positions marked. *B*, ITC binding curve showing direct interaction between the CBY1 FL and FAM92A BAR domain with *K*_*d*_ around 0.15 μM. *C*, ITC binding curve showing reduced affinity of FAM92A BAR and CBY1-NT (*K*_*d*_ ∼3.6 μM). *D*, the table summarizing the ITC-based and the BLI-based measurements of the binding affinities between variants of CBY1 and FAM92A BAR, demonstrating that their dimerization synergistically drives high-affinity binding. *E*, Strep-Tactin pull-down assays confirming that dimerization of CBY1 is essential for high-affinity FAM92A BAR binding. Assays used purified Trx-Strep-tagged FAM92A BAR and His-tagged CBY1 variants (MBP-His-tagged CBY1-FL, MBP-His-tagged CBY1-4E, and GB1-His-tagged CBY1-NT-GCN4), analyzed by SDS-PAGE with Coomassie blue staining. BAR, Bin/Amphiphysin/Rvs; BLI, biolayer interferometry; FAM92A, family with sequence similarity 92A; ITC, isothermal titration calorimetry.

Beyond dimerization and membrane binding, the FAM92A BAR domain is well known for mediating interactions with CBY1 to regulate ciliogenesis (18). CBY1, a 15-kDa protein, comprises an unstructured N-terminal (NT) region (residues 1–70) and a C-terminal CC domain (residues 61–127) (Fig. 5*A*1). SEC–MALS analysis revealed that recombinant maltose-binding protein (MBP)–tagged full-length CBY1 (CBY1 FL) forms a stable dimer, likely mediated by its CC domain (Fig. S3*A*). Isothermal titration calorimetry (ITC) and biolayer interferometry (BLI) confirmed a strong, direct interaction between CBY1-FL and FAM92A BAR, with dissociation constants (*K*_*d*_) of ∼0.15 μM (ITC) and ∼0.25 μM (BLI) (Figs. 5*B* and S4*A*). To identify the CBY1 region responsible for binding, we expressed recombinant Trx-tagged CBY1 NT and CBY1 CC fragments. As predicted, SEC–MALS showed CBY1 NT as a monomer and CBY1 CC as a dimer (Fig. S3, *B* and *C*). While CBY1 NT retained binding to FAM92A BAR, its affinity was markedly reduced (*K*_*d*_ ∼3.6 μM by ITC, ∼10 μM by BLI), and CBY1 CC exhibited no detectable interaction (Figs. 5, *C* and *D*, and S4*B*).

Given that both FAM92A BAR and CBY1 FL are dimers, we hypothesized that their high affinity results from synergistic enhancement by their respective dimerization. To test this, we introduced the aforementioned dimer-disrupting mutations into FAM92A BAR (L120K/I257Q, Y127K/F256Q, and L113K/H264E). ITC detected negligible binding (Fig. 5*D*). Next, we disrupted CBY1 dimerization by mutating four conserved leucine residues (L77, L84, L91, and L98) in its CC domain to glutamic acid (denoted as CBY1 4E) (Fig. 5*A*2). SEC–MALS confirmed the success of this mutagenesis design that CBY1 4E exists as a monomer (Fig. S5*A*). ITC and BLI revealed that CBY1 4E bound FAM92A BAR at levels comparable to CBY1 NT (*K*_*d*_ ∼2.8 and 5.6 μM, respectively) (Figs. 5*D* and S4*C*). To further confirm that CBY1 CC contributes solely through dimerization synergy, we replaced the native CC with the well-characterized GCN4 CC (33) (termed CBY1 NT-GCN4) (Figs. 5*A*3 and S5*B*). As expected, ITC and BLI restored binding affinity to the submicromolar range (Figs. 5*D* and S4*D*). We further showed that purified Strep-FAM92A BAR can successfully pull down full-length MBP-CBY1. While the pull-down amount of MBP-CBY1 4E was significantly reduced and the pull-down amount of GB1-CBY1 NT-GCN4 was comparable to the WT (Fig. 5*E*). These results demonstrate that FAM92A BAR directly interacts with CBY1, with their dimerization cooperatively driving high-affinity binding.

## Discussion

In this study, we resolved the high-resolution crystal structure of the mouse FAM92A BAR domain at 2.2 Å and conducted structure-guided biochemical analyses to delineate the structural determinants of its dimerization and membrane binding. The antiparallel dimer, stabilized by an extensive interface, adopts a crescent shape typical of classical BAR domains. We also identified several clusters of positively charged residues in the concave surface responsible for lipid binding, yet whether this curvature-sensing capability influences ciliary morphology or other processes like mitochondrial ultrastructure and neuronal membrane dynamics remains unclear. These findings, complemented by the recently reported human FAM92A1 BAR structure (23), establish a molecular framework for elucidating FAM92A’s roles in membrane remodeling, including ciliogenesis, mitochondrial morphology maintenance, and neuronal function.

We biochemically delineated the FAM92A BAR–CBY1 interaction, identifying CBY1’s NT region as the primary binding site, with its C-terminal CC-mediated dimerization synergistically boosting affinity (more than 10-fold increase). Although AlphaFold predictions align with this model, pinpointing CBY1’s NT 22 residues as critical for binding (18, 34), the structural basis of this interaction remains computationally derived, necessitating high-resolution experimental structures (*e*.*g*., cocrystallization) for definitive validation. Notably, CBY1’s NT is enriched with positively charged residues, and AlphaFold suggests their proximity to FAM92A’s concave surface, hinting that CBY1 binding may enhance FAM92A’s membrane interactions, a possibility meriting further investigation. Sequence analysis indicates this binding mode may be conserved across the CBY family, with CBY3 sharing NT similarity and a CC domain similar to CBY1’s (26), though experimental validation is needed. Likewise, while *in vitro* data underscore dimerization synergy, the *in vivo* relevance requires confirmation. In addition, the conserved CBY1 CC may interact with other ciliary proteins (*e*.*g*., CEP164 (35), DZIP1 (36)) beyond its synergistic role, emphasizing the need for comprehensive interaction studies to elucidate its broader network. Furthermore, the functional binding partners of FAM92A mediating its mitochondrial and neuronal roles remain to be identified.

FAM92B, another FAM92 family member, shares >50% sequence identity and ∼75% similarity with FAM92A, suggesting functional overlap. Studies indicate that FAM92B, like FAM92A, forms homodimers, heterodimerizes with FAM92A, binds membranes, and interacts with CBY1 (18). AlphaFold-based predictions further reveal that the FAM92B dimer closely resembles our FAM92A BAR crystal structure, with nearly identical CBY1-binding sites, supporting a high degree of redundancy. However, FAM92B cannot fully compensate for FAM92A loss, as FAM92A knockout or knockdown in animal models induces distinct abnormalities, despite the presence of FAM92B. This divergence may stem from differential expression across developmental stages or tissues; for instance, the Human Protein Atlas database (https://www.proteinatlas.org/) indicates FAM92A enrichment in both male and female tissues, whereas FAM92B appears absent in male tissues. Alternatively, it could reflect unique functions beyond their shared properties, such as whether FAM92B localizes to mitochondria and modulates their morphology like FAM92A. These aspects remain to be elucidated.

## Experimental procedures

### Constructs and protein purification

The coding sequences of mouse FAM92A (GenBank: NP_080834.3, amino acids 1–355) and mouse CBY1 (GenBank: NP_082910.1, amino acids 1–127) were PCR amplified from the mouse complementary DNA library. Yeast GCN4 (GenBank: NP_010907.3, amino acids 249–281: RMKQLEDKVEELLSKNYHLENEVARLKKLVGER) was obtained through gene fragment homologous recombination. The following constructs were cloned into modified pET-32M vectors with NT Trx-His6, MBP-His6, Trx-Strep2, or MBP-Strep2 tags, and an HRV-3C protease cleavage site: mouse FAM92A (amino acids 1–355), mouse FAM92A BAR (amino acids 70–283), mouse CBY1 (amino acids 1–127), mouse CBY1 NT (amino acids 1–70), mouse CBY1 CC (amino acids 61–127), and yeast GCN4 (amino acids 249–281). The Trx-CBY1-NT-GCN4 fusion protein was generated by homologous recombination, fusing the yeast GCN4 CC domain to the C terminus of Trx-tagged CBY1-NT (residues 1–70) in a modified pET-32M vector. All constructs were verified by DNA sequencing. All point mutations of FAM92A and CBY1 used in this study were created using the standard PCR-based mutagenesis method and confirmed by DNA sequencing. All proteins were expressed in *E*. *coli* BL21 (DE3; Invitrogen, catalog no.: C600003). Proteins with NT His6 tags were purified using a Ni^2+^–NTA Sepharose 6 Fast Flow column (Cytiva, catalog no.: 17531803), whereas those with two Strep tags were purified using a Strep-Tactin Sepharose (IBA, catalog no.: 205090) column. Subsequently, preparative and SEC were performed using HiLoad 26/600 Superdex 75 pg (Cytiva, catalog no.: 28989334) or HiLoad 26/600 Superdex 200 pg columns (Cytiva, catalog no.: 28989336) with a buffer containing 50 mM Tris (pH 7.5), 1 mM DTT, 1 mM EDTA, and 100 mM NaCl.

### Size-exclusion chromatography coupled with multiangle light scattering

Protein samples (typically 200 μl at a concentration of 70 μM pre-equilibrated with column buffer) were injected into an ÄKTA FPLC system with a Superose 12 10/300 GL column (Cytiva, catalog no.: 17517301) using the column buffer of 50 mM Tris (pH 7.5), 1 mM DTT, 1 mM EDTA, and 100 mM NaCl. The chromatography system was coupled to a MALS system equipped with a three-angle static light scattering detector (miniDawn; Wyatt) and a differential refractive index detector (Optilab; Wyatt). The elution profiles were analyzed using the ASTRA 7 software (Wyatt).

### Crystallography

Crystals of the FAM92A BAR domain (in 50 mM Tris [pH 7.5], 1 mM DTT, 1 mM EDTA, and 100 mM NaCl buffer) were obtained by sitting drop vapor diffusion methods at 16 °C. The crystals were grown in buffer containing 15% w/v polyethylene glycol 3350, 10 mM magnesium chloride hexahydrate, and 5 mM nickel (II) chloride hexahydrate, pH 7.0 and soaked in crystallization solution containing additional 20% glycerol for cryoprotection. Diffraction data were collected at the Shanghai Synchrotron Radiation Facility BL19U1 at 100 K. Data were automated processed and scaled using the aquarium pipeline (37).

The FAM92A BAR domain structure was solved by molecular replacement in Phaser (38), using the AlphaFold2-generated model of mouse FAM92A as the search template (31). Initial phases were significantly improved through autobuilding with Buccaneer (https://www.ccp4.ac.uk/) (39). The structure was refined through iterative cycles of manual adjustment in COOT (https://www2.mrc-lmb.cam.ac.uk/Personal/pemsley/coot/) (40) and refinement in PHENIX (https://www.phenix-online.org/) (41). Model quality was assessed using MolProbity (42), with final refinement statistics summarized in Table 1. Structural figures were generated using PyMOL (http://www.pymol.org).

### Pull-down assay

For pull-down assays, Trx-Strep-tagged FAM92A BAR and CBY1 proteins (MBP-tagged CBY1-FL, MBP-tagged CBY1-4E, or GB1-tagged CBY1-NT-GCN4) were purified individually by SEC (see constructs and protein purification part). Purified proteins were adjusted to equimolar concentration using buffer containing 50 mM Tris (pH 7.5), 1 mM DTT, 1 mM EDTA, and 100 mM NaCl. Reactions (500 μl) were prepared by mixing 250 μl of a CBY1 variant with 250 μl of Trx-Strep-tagged FAM92A BAR and 20 μl of Strep-Tactin beads (IBA, catalog no.: 205090). The mixture was incubated at 4 °C for 30 min. After incubation, the beads were washed several times with buffer. Strep-tagged fusion proteins and their interacting partners were analyzed by SDS-PAGE, visualized with Coomassie blue staining, and imaged using a gel imaging analyzer (SINSAGE, ChampGel 5000 Plus; SAGECREATION). Three independent experiments were performed.

### ITC assay

ITC measurements were performed on a MicroCal PEAQ-ITC calorimeter (Malvern) at 25 °C. All proteins were dissolved in a buffer containing 50 mM Tris (pH 7.5), 1 mM DTT, 1 mM EDTA, and 100 mM NaCl. High concentrations (200–300 μM) of each binding partner tested in this study, including MBP-CBY1 FL, Trx-CBY1 NT, MBP-CBY1 4E, and CBY1 NT-GCN4 proteins, were loaded into the syringe, whereas the corresponding FAM92A BAR protein or its dimerization mutants (100–120 μM) were placed in the cell. Each titration point was obtained by injecting a 10 μl aliquot of the syringe protein into the cell containing various protein samples at 120 s intervals to ensure complete return of the titration peak to baseline. The titration data were analyzed using the MicroCal PEAQ-ITC Analysis software (Malvern) and fitted with a one-site binding model.

### BLI assay

BLI assays were performed using a Gator Pilot biolayer interferometry instrument (Gator Bio). Trx-Strep-tagged FAM92A BAR, MBP-His-tagged CBY1 FL, Trx-His-tagged CBY1 NT, MBP-His-tagged CBY1 4E, and GB1-His-tagged CBY1 NT-GCN4 were expressed and purified as described (see constructs and protein purification part). Kinetic measurements were conducted in interaction buffer (PBS with 0.02% Tween-20 and 0.2% bovine serum albumin). Trx-Strep-tagged FAM92A BAR was diluted to 1 μM. MBP-His-tagged CBY1 FL and GB1-His-tagged CBY1-NT-GCN4 proteins were diluted to 20 μM, 10 μM, 5 μM, 2.5 μM, 1250 nM, and 625 nM in the interaction buffer. Similarly, the MBP-His-tagged CBY1 4E and Trx-His-tagged CBY1 NT proteins were diluted in the interaction buffer to final concentrations of 2 μM, 1 μM, 500 nM, 250 nM, 125 nM, and 62.5 nM. Strep-Tactin XT probes (Gator; part number: 160033) were hydrated in the interaction buffer for 10 min before use. Assays involved a 90 s baseline in interaction buffer, followed by 60 s loading of Strep-tagged FAM92A BAR onto the probes. Probes were immersed in His-tagged protein solutions for 120 s to measure association and then transferred to the interaction buffer for dissociation for 200 s. A reference probe in the interaction buffer alone was used for background subtraction. Reference-subtracted data were fitted to a 1:1 binding model using Gator Part11 software (21 CFR Part 11 Compliant BLI software, https://www.gatorbio.com/products/software/).

### Brain liposome–binding assay

Bovine brain lipid extract (Folch Fraction I; Sigma, catalog no.: B1502) was dissolved in chloroform to 10 mg/ml and stored at −20 °C. For each experiment, 1 mg total lipid extract was dried under a nitrogen stream followed by 1 h vacuum desiccation to remove residual chloroform. Dried lipids were hydrated in 400 μl HEPES-buffered saline (20 mM HEPES [pH 7.5] and 100 mM NaCl) with extensive pipetting and vortexing for 1 h. Liposomes were formed *via* sonication (Branson 250 Sonifier, duty circle: 50%, output control: 5) until the emulsion gradually became semitransparent. The liposome concentration was estimated by the initial input, assuming negligible loss during preparation.

For liposome binding, 10 μM FAM92A BAR (or its mutants) was incubated with 2 mg/ml liposomes in HEPES-buffered saline (50 μl total volume) for 10 min at room temperature. Protein-only samples (no liposomes, as controls) were processed identically to assess nonspecific pelleting. Mixtures and controls were ultracentrifuged at 200,000*g* for 20 min at 4 °C using a Beckman TLA120.1 rotor to pellet liposomes. The supernatant was collected as sample S, and the pellet was resuspended in 50 μl HEPES-buffered saline. Supernatant and pellet fractions were analyzed by SDS-PAGE with Coomassie blue staining. Each experiment was repeated three times. The band intensity on SDS-PAGE gel was quantified by ImageJ (https://imagej.net/ij/). Pellet percentages were reported as mean ± SD.

## Data availability

The atomic coordinates and the structure factors of mouse FAM92A BAR domain structure have been deposited to the PDB under the accession code 9UHD.

## Supporting information

This article contains supporting information.

## Conflict of interest

The authors declare that they have no conflicts of interest with the contents of this article.
