# Supplementary material for: Dimerization of the BAR domain–containing protein FAM92A modulates lipid binding and interaction with CBY1
Source: J Biol Chem. 2025 Jun 6;301(7):110346. doi: 10.1016/j.jbc.2025.110346 (PMC12269497; doi:10.1016/j.jbc.2025.110346)
Supplement: Supplementary Material [file mmc1.docx]

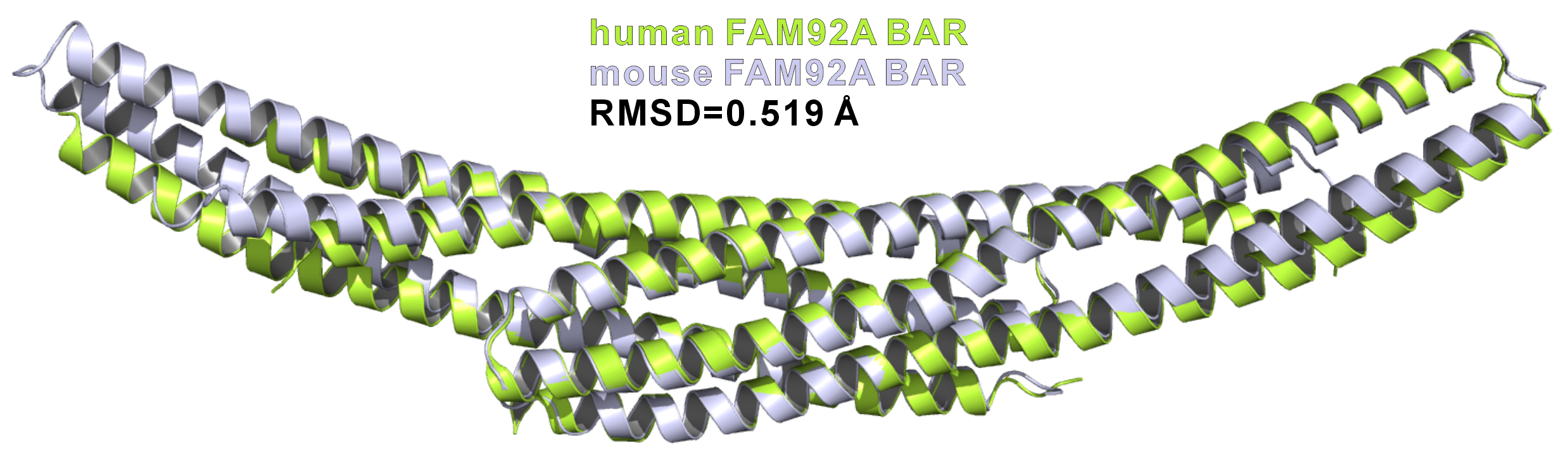


**Supplementary Figure 1. Structural Comparison of Mouse and Human FAM92A BAR Domains.**

Superposition of the mouse FAM92A BAR domain structure (light blue, 2.2 Å resolution) with the human FAM92A1 BAR domain (green, PDB: 8CEG), revealing high structural conservation (RMSD 0.519 Å). The superposition was generated by aligning the mouse and human dimeric FAM92A BAR domain structures using the cealign algorithm in PyMOL.


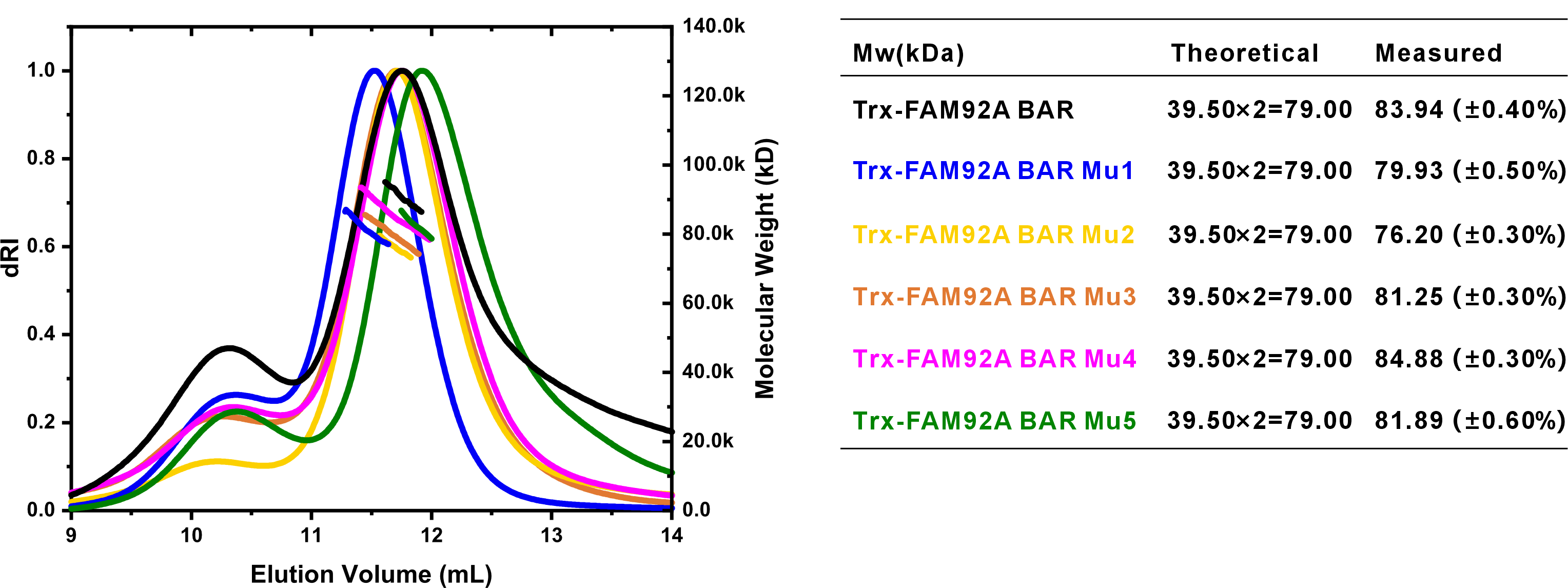


**Supplementary Figure 2.** **SEC-MALS Analysis of Charge-Altered Mutants of FAM92A BAR.**

SEC-MALS data for FAM92A BAR charge-altered mutants, showing SEC-MALS profiles (left) and measured molecular weights (right). These results indicate that mutations of positively charged residues on the concave surface do not disrupt homodimer formation. The measured molecular weights are expressed as M_n_ (±x%).


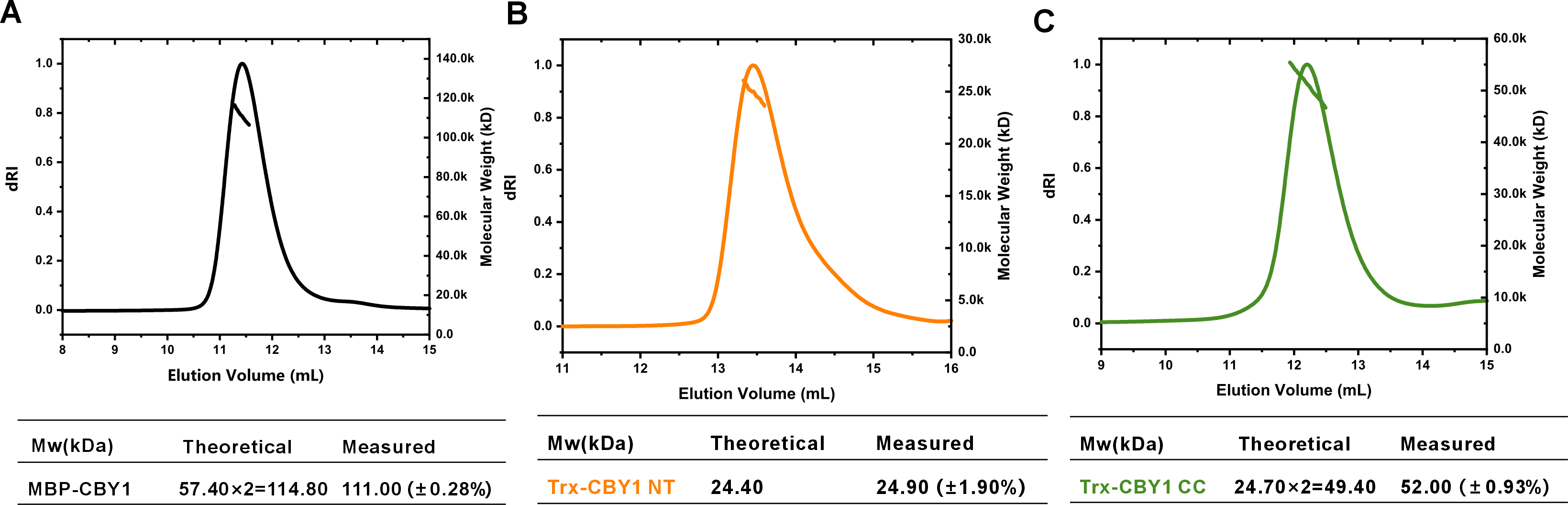


**Supplementary Figure 3.** **Oligomeric States of CBY1 Fragments by SEC-MALS**

SEC-MALS profiles showing dimerization of MBP-tagged full-length CBY1 (A) and CBY1 coiled-coil domain (C), and monomeric state of CBY1 N-terminal region (B). The measured molecular weights are expressed as M_n_ (±x%).


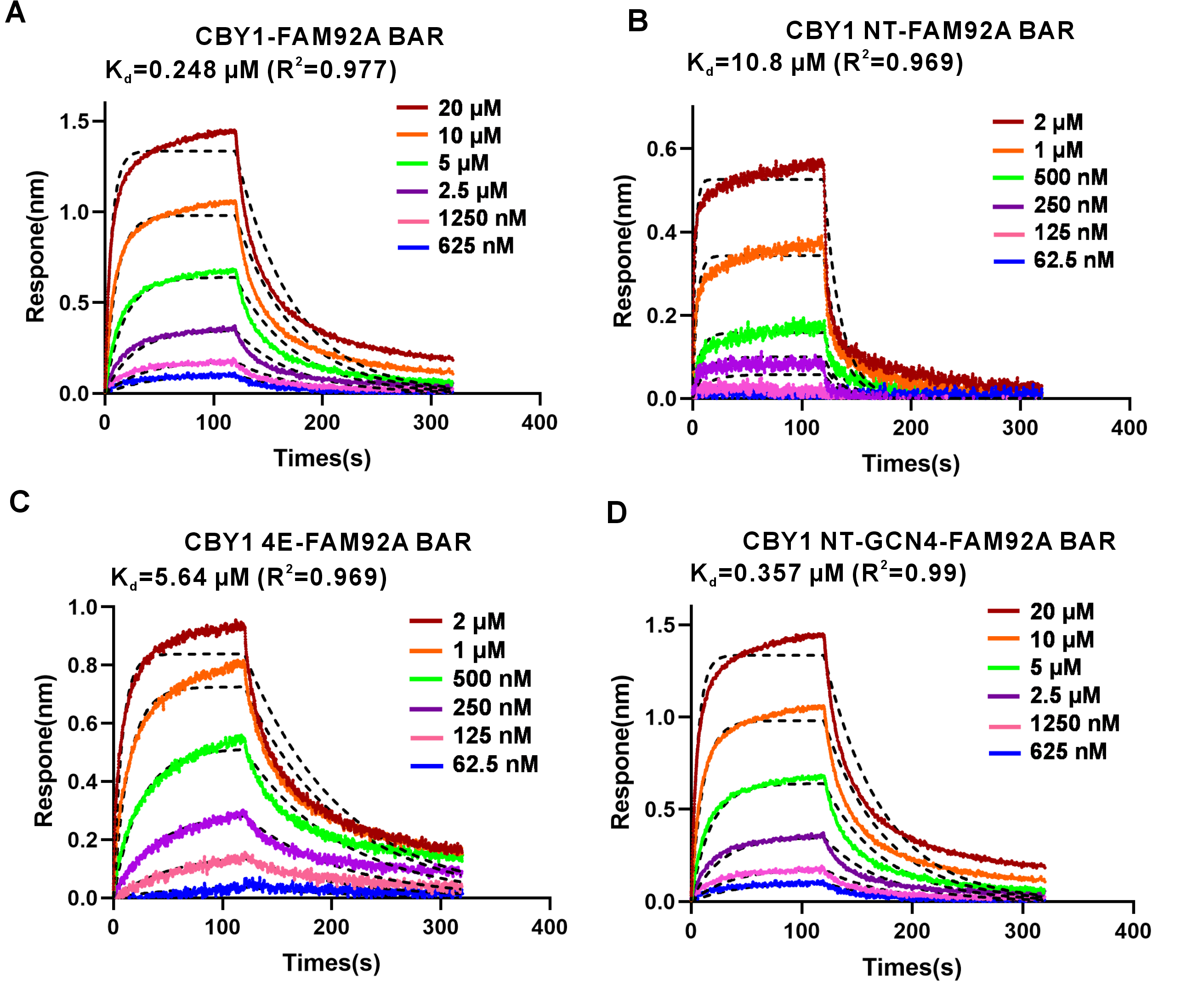


**Supplementary Figure 4.** **BLI Analysis of FAM92A BAR and CBY1 Interactions.**

BLI binding curves and derived dissociation constants for FAM92A BAR interactions with CBY1 variants: (A) full-length CBY1 (CBY1-FL), (B) CBY1 NT, (C) CBY1-4E mutant, and (D) CBY1-NT-GCN4. Results are consistent with ITC data. The FAM92A BAR was immobilized on the sensor tips, and CBY1 and its variants were used as the analyte in solution at the indicated concentrations.


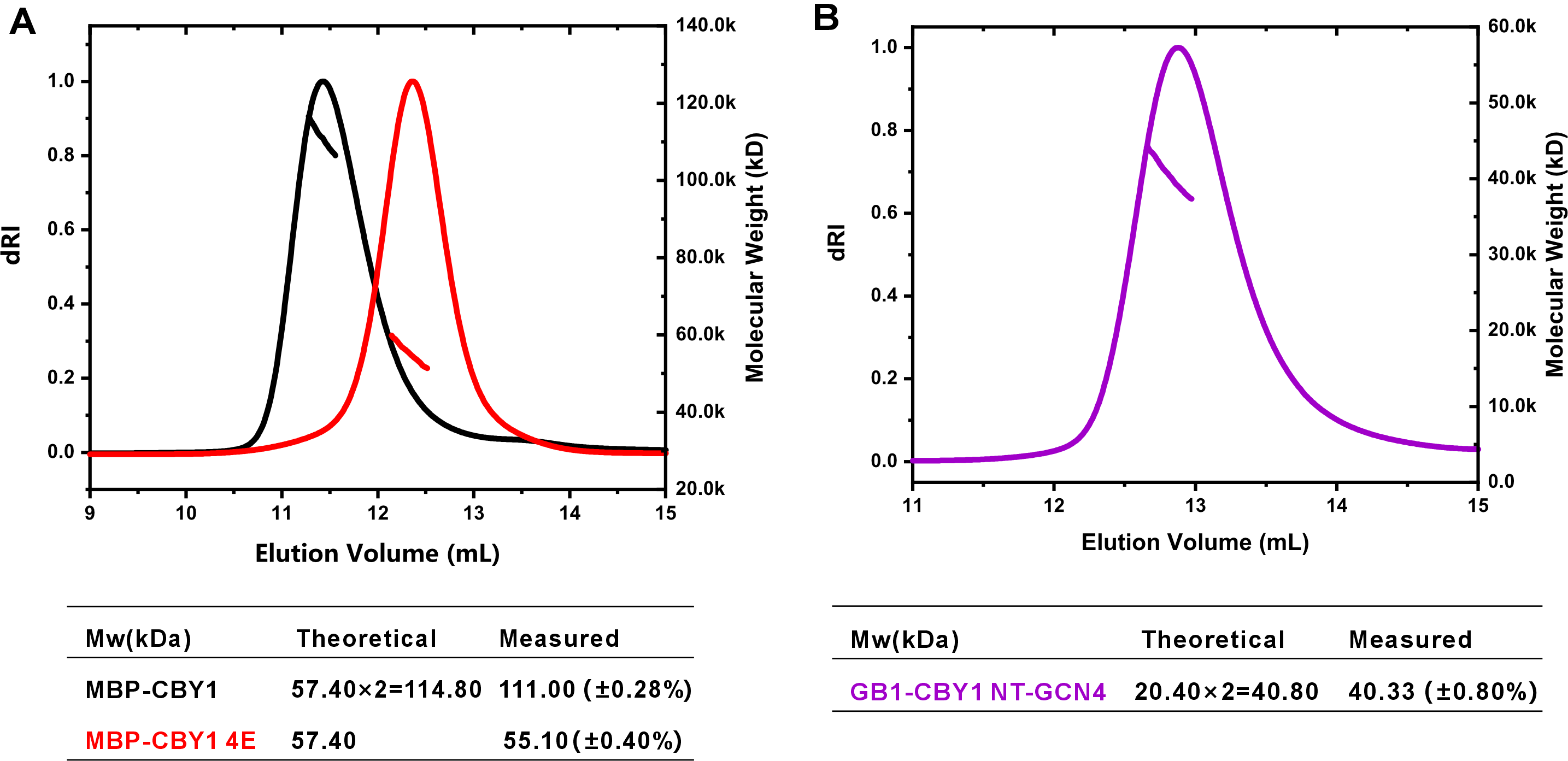


**Supplementary Figure 5.** **SEC-MALS Analysis of CBY1-4A and CBY1-NT-GCN4**

SEC-MALS profiles showing that CBY1-4E (L77E, L84E, L91E, L98E; A) is monomeric, while CBY1-NT-GCN4 (N-terminal region fused to GCN4 coiled-coil; B) is dimeric. The measured molecular weights are expressed as M_n_ (±x%).
